# “Weibing” in traditional Chinese medicine—biological basis and mathematical representation of disease-susceptible state

**DOI:** 10.1016/j.apsb.2025.03.009

**Published:** 2025-03-08

**Authors:** Wanyang Sun, Rong Wang, Shuhua Ouyang, Wanli Liang, Junwei Duan, Wenyong Gong, Lianting Hu, Xiujuan Chen, Yifang Li, Hiroshi Kurihara, Xinsheng Yao, Hao Gao, Rongrong He

**Affiliations:** a Institute of Traditional Chinese Medicine and Natural Products, College of Pharmacy/Guangdong Engineering Research Center of Traditional Chinese Medicine & Disease Susceptibility/Guangdong Engineering Research Center of Traditional Chinese Medicine & Health Products/International Cooperative Laboratory of TCM Modernization and Innovative Drug Development of Chinese Ministry of Education (MOE)/Guangdong Province Key Laboratory of Pharmacodynamic Constituents of TCM and New Drugs Research/State Key Laboratory of Bioactive Molecules and Druggability Assessment, Jinan University, Guangzhou 510632, China; b State Key Laboratory of Quality Research in Chinese Medicine, Macau University of Science and Technology, Macau 999078, China; c College of Information Science and Technology, Jinan University, Guangzhou 510632, China; d Faculty of Data Science, City University of Macau, Macau 999078, China; e Medical Big Data Center, Guangdong Provincial People’s Hospital, Guangdong Academy of Medical Sciences, Guangzhou 510632, China; f The Data Center, Wuhan Children’s Hospital (Wuhan Maternal and Child Healthcare Hospital), Tongji Medical College, Huazhong University of Science and Technology, Wuhan 430016, China

**Keywords:** Weibing, Disease-susceptible state, Traditional Chinese medicine, Preventive medicine, Mathematical modeling

## Abstract

“Weibing” is a fundamental concept in traditional Chinese medicine (TCM), representing a transitional state characterized by diminished self-regulatory abilities without overt physiological or social dysfunction. This perspective delves into the biological foundations and quantifiable markers of Weibing, aiming to establish a research framework for early disease intervention. Here, we propose the “Health Quadrant Classification” system, which divides the state of human body into health, sub-health, disease-susceptible state, and disease. We suggest the disease-susceptible stage emerges as a pivotal point for TCM interventions. To understand the intrinsic dynamics of this state, we propose laboratory and clinical studies utilizing time-series experiments and stress-induced disease susceptibility models. At the molecular level, bio-omics technologies and bioinformatics approaches are highlighted for uncovering intricate changes during disease progression. Furthermore, we discuss the application of mathematical models and artificial intelligence in developing early warning systems to anticipate and avert the transition from health to disease. This approach resonates with TCM’s preventive philosophy, emphasizing proactive health maintenance and disease prevention. Ultimately, our perspective underscores the significance of integrating modern scientific methodologies with TCM principles to propel Weibing research and early intervention strategies forward.

## Graphical abstract

This paper explores the concept of “Weibing” in TCM, introduces the disease-susceptible stage as a critical period for “Weibing” research, and discusses the use of mathematical models and AI to develop early warning systems for identifying disease-susceptible conditions in clinical settings.

**Figure:**
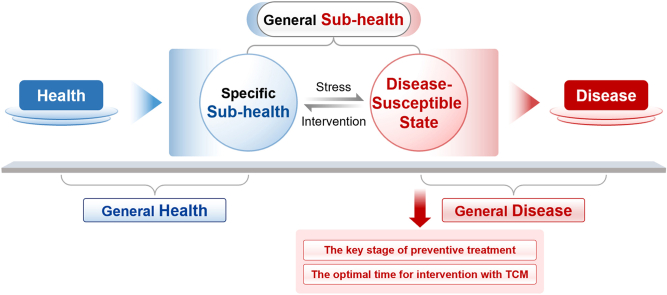

## 1 Introduction

“Weibing” (Chinese expression in Supporting Information), a pivotal concept in traditional Chinese medicine (TCM), refers to a health status characterized by a subtle decline in an individual’s innate abilities for self-organization, adaptation, and repair, without significantly compromising physiological or social functions. The traditional Weibing theory revolves around four core principles: forestalling disease onset, arresting its progression, mitigating exacerbation, and averting recurrence. These principles are deeply entrenched in preventive medicine, advocating proactive health maintenance strategies to delay or avert the onset of illness. Specifically, preventive actions should be initiated during healthy period to safeguard against sickness. Upon disease diagnosis, efforts must concentrate on halting its progression and preventing deterioration. In cases where a disease has advanced, the focus shifts to containing further decline. Post-treatment, preventive steps are vital to prevent recurrence or the emergence of secondary conditions. This approach underscores TCM’s “prevention first” philosophy, where preemptive treatment aligns with broader preventive medicine principles, advancing the objectives of disease prevention and management. Despite its centuries-old theoretical foundation, the biological mechanisms and quantifiable markers of Weibing remain elusive in TCM. Thus, establishing a robust research framework to explore the biological underpinnings of Weibing, develop mathematical models to identify critical states, and implement early warning systems is imperative.

## 2 Disease-susceptible state is the critical stage for intervention before disease onset

TCM distinguishes itself from modern medical paradigms by its emphasis on the concept of “syndrome”, a nuanced integration of symptoms and signs indicative of specific bodily imbalances or disorders^1^. Examples include heat syndrome and cold syndrome, each embodying distinct physiological patterns^2^. A notable example is the “Shanghuo” syndrome^3^, a subtype of heat syndrome, triggered by multifaceted factors including psychological stress, insomnia, dietary indiscretions, and excessive smoking^4^, ^5^, ^6^. The manifestation of “Shanghuo” spans a diverse array of symptoms, varying from generalized expressions like redness, pain, and fever to more localized conditions such as acne outbreaks and nosebleeds. Notably, this syndrome does not correspond directly to a single, definitive disease entity but rather serves as an early indicator of potential health challenges that may emerges^7^. As a result, the absence of suitable diagnostic models tailored specifically for “Shanghuo” poses challenges in assessing the efficacy of TCM interventions aimed at addressing this condition. Consequently, research and development in this area are crucial to enhance understanding and improve treatment outcomes for individuals experiencing TCM syndromes.

**Figure 1:**
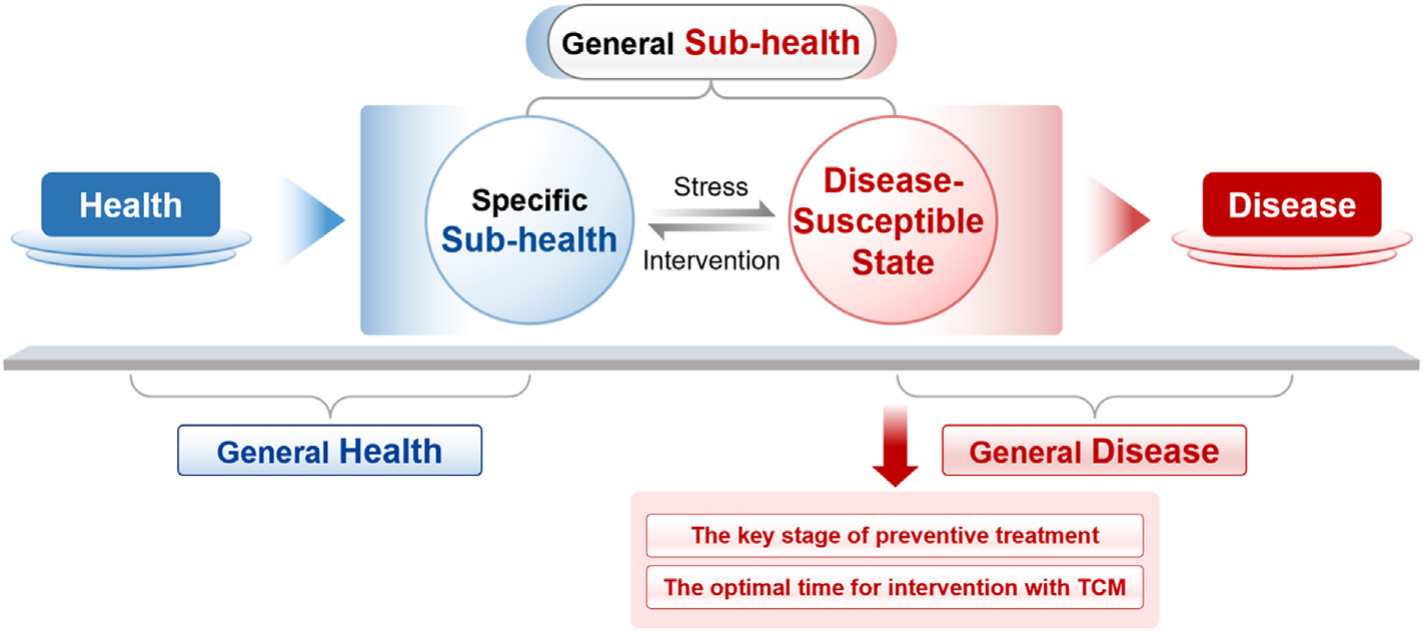
Precise quadrant division of health states. This figure illustrates a precise categorization of health states into four distinct quadrants, each representing varying degrees of physiological well-being. The first quadrant, labeled “General Health”, encompasses a state of overall well-being, free from overt organic changes and typically not requiring pharmacological interventions. The adjacent quadrant, “Specific Sub-health”, also falls under the broader category of “General Health”, characterized by mild imbalances or discomforts without definitive disease markers. Moving towards the lower left, the second quadrant, “Specific Sub-health”, transitions towards “Disease-Susceptible State”, a state of increased vulnerability where individuals may exhibit subtle symptoms or imbalances, not yet constituting a full-fledged disease. Both of these quadrants are grouped under “General Sub-health”, highlighting the absence of overt disease indications. The final two quadrants represent a progression towards more severe conditions. The “Disease-Susceptible State” makes a critical juncture where early intervention can significantly impact the progression towards a definitive disease. This state is distinct from generalized sub-health and underscores the importance of early intervention. The last quadrant, “Disease”, encompasses varying degrees and types of pathological changes that often necessitate targeted medical interventions, including specific pharmacological treatments.

To address the complexity of evaluating TCM treatments aimed at mitigating “Shanghuo” and similar critical states, we propose a refined classification system for health-disease states, grounded in TCM principles. This framework encapsulates four stages: health, sub-health, disease susceptible state, and disease (Fig. 1). Health and a special subcategory of sub-health, devoid of overt pathological markers, comprise the general health stage, necessitating no medication. Conversely, the subsequent stages—specific sub-health and disease-susceptible state—though not diagnostic of specific diseases, represent a generalized sub-health category marked by subtle physiological changes. Crucially, the disease-susceptible state, represents a critical threshold where the body teeters on the brink of full-blown illness. This refined pathological state surpasses the ambiguities of generalized sub-health, highlighting its strategic importance as a pivot for early intervention. In this delicate balance, the body exhibits heightened sensitivity to external stressors, portending a swift descent into disease if left unchecked. As such, disease-susceptible state represents a vital nexus in the TCM paradigm of “preventive treatment of disease,” ideally suited for TCM’s adaptive regulatory strategies.

Overall, Fig. 1 provides a visual representation of the continuum of health states, emphasizing the pivotal role of the disease-susceptible stage in preventive medicine and highlighting the opportunities for early intervention, particularly within the framework of TCM. The timely application of TCM interventions during this critical phase can significantly influence the trajectory of impending illness, averting the onset of full-blown disease and restoring balance to the body. Conversely, the absence of appropriate measures during this window may precipitate a rapid deterioration into disease, exacerbating treatment challenges. Thus, recognizing and addressing disease-susceptible state, as embodied in TCM’s nuanced classification system, holds the key to optimizing health outcomes and preventing the onset of avoidable illnesses.

## 3 Laboratory and clinical studies for understanding disease-susceptible state

The intricate dynamics of disease progression underscore the imperative for time-series experiments in elucidating and modeling these complex processes^8^^,^^9^. In the laboratory setting, these experiments on cellular and animal models offer a unique window into the temporal evolution of diseases, allowing researchers to discern patterns and trends over time. While offering unparalleled insights, such studies confront challenges, including sample grouping limitations and the demands of extended follow-up, especially for chronic diseases characterized by lengthy onset periods. To overcome these obstacles, we previously employ various stress factors and classic disease inducers to expedite the transition of models into critical disease states, particularly among those in suboptimal health. By employing psychological stress and classic disease-inducing factors, researchers have successfully constructed disease-susceptibility models for major diseases, including viral infections^10^, ^11^, ^12^, neurodegenerative diseases^13^, ^14^, ^15^, ^16^, ^17^, tumors^18^, ^19^, ^20^, ^21^, metabolic diseases^22^^,^^23^, immune diseases^24^^,^^25^, and cardiovascular diseases^26^^,^^27^. These models allow the evaluation of the efficacy of both TCM formula^28^, ^29^, ^30^, ^31^, ^32^ and active compounds^33^, ^34^, ^35^, ^36^, ^37^, ^38^. The disease-susceptibility model makes it easier for experimental animals to be in a disease-susceptible state or critical transformation state, at which point TCM intervention can effectively delay or even reverse disease progression.

Translating these laboratory findings into clinical practice, however, necessitates the acquisition of high-quality clinical data that meticulously documents disease progression. Longitudinal studies, particularly cohort, interventional longitudinal, and longitudinal case-series studies, offer robust methodologies for monitoring disease evolution over time. Cohort studies, for instance, prospectively follow individuals to uncover disease development patterns, as exemplified by research on chronic kidney disease progression^39^. Interventional longitudinal studies, by exposing healthy subjects to pathogens and tracking symptomology and gene expression over time, yield invaluable insights into the temporal profiles of host responses^40^. Meanwhile, longitudinal case-series studies meticulously track individual disease trajectories, pinpointing critical intervention junctures despite their data-intensive requirements. Recognizing the constraints associated with traditional longitudinal studies, computational methods leveraging cross-sectional data have emerged as valuable complements^41^. These techniques reconstruct time-series models from cross-sectional snapshots, enabling the identification of distinct disease states and their temporal progression, even in the absence of direct time-series data. This strategy has demonstrated remarkable success in modeling glaucoma, breast cancer, Parkinson’s disease, and myelodysplastic syndromes^42^, ^43^, ^44^, ^45^, revolutionizing our comprehension of disease mechanisms and intervention strategies.

In conclusion, a multifaceted approach that integrates laboratory experimentation, clinical studies, and computational modeling holds immense promise for advancing our understanding of disease-susceptible states and facilitating the development of targeted interventions that can significantly impact public health outcomes.

## 4 The macroscopic and microscopic aspects of disease-susceptible state

The physiological characteristics of a patient can be primarily assessed through two distinct lenses: macroscopic approaches, encompassing clinical signs and imaging, and microscopic approaches, focusing on molecular data. For macroscopic analysis, both modern medicine and TCM offer diagnostic methodologies. Modern medicine primarily utilizes advanced clinical examination and imaging techniques to discern health and disease states^46^. In contrast, TCM relies on the “Four Diagnostic Methods” - observation, auscultation, interrogation, and palpation - to analyze the body’s response, dynamics, and changes throughout the disease process^47^. This leads to the concept of “syndrome,” which captures a specific stage in disease progression and the individual’s internal and external environment^48^^,^^49^, manifested through symptoms like tongue and pulse analysis, along with observations of shape, color, and vitality^50^^,^^51^. Notably, the “syndrome” concept has the potential to indicate a disease-susceptible state to a certain degree^52^. For instance, in the stage of transitioning from hepatitis B carrier to illness, TCM can diagnose imbalances such as Yin-Yang disharmony and Qi-blood disturbance, along with specific syndromes like liver-Qi stagnation, liver fire, Qi stagnation with blood stasis, etc.^53^

**Figure 2:**
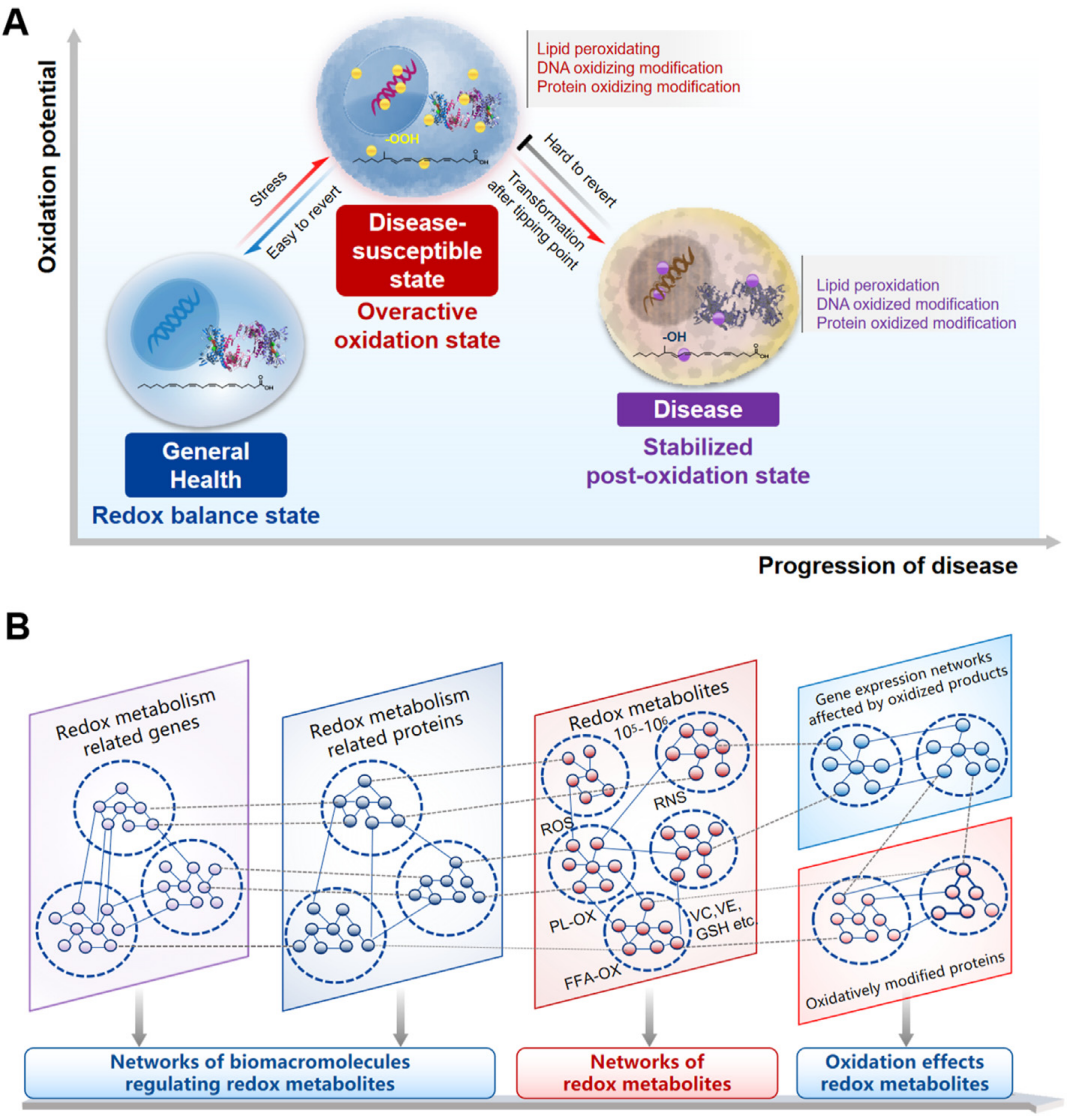
Perturbation of redox balance in disease-susceptible state and its impact on small molecule-macromolecule. (A) The disease-susceptible state at the cellular level. In the healthy state, the body’s oxidation-reduction system functions normally, and lipid oxidation is controlled within the threshold range. In contrast, the disease-susceptible state is characterized by highly active oxidation reactions in the body, with the chain reaction of lipid free radicals rapidly amplifying and producing excessive and highly active lipid oxidation products. Consequently, intracellular genes and proteins become oxidized and modified by lipid oxidation. In the disease state, the intracellular oxidation reaction is not active, and the lipids, proteins, and genes of the cells are in a relatively stable state after oxidative modification. (B) The small molecule and macromolecule interaction network in the disease-susceptible state. This state is postulated to be characterized by a unique biomarker profile, primarily comprising intracellular oxidized small molecules, lipid peroxidation products, and products resulting from oxidative modifications of genes and proteins. This hypothesis is grounded in the extensive literature exploring the field, suggesting that these biomarkers may serve as crucial indicators of impending disease.

At the microscopic level, disease progression is characterized by continuous changes and dynamic interactions among biomolecules, including genes, proteins, and metabolites^54^^,^^55^. To attain a comprehensive understanding of the dynamic molecular changes occurring within the body during disease progression, bio-omics technology, combined with bioinformatics methods, offers an inclusive perspective by examining the microscopic aspects of the body and analyzing vast quantities of molecular data derived from living organisms^56^^,^^57^. This approach encompasses various fields, such as genomics, transcriptomics, proteomics, metabolomics, and epigenomics. Our recent research has indicated that the body can develop into a disease-susceptible state under stress conditions such as emotions, diet, and environment^7^^,^^11^^,^^58^^,^^59^. This state is marked by elevated levels of free radicals which can disrupt the cell’s redox balance, leading to an overactive intracellular oxidation reaction (Fig. 2A)^60^. This, in turn, causes severe lipid peroxidation within cells, significantly modifying proteins and genes through oxidized lipids. Once the oxidation level surpasses a critical threshold, it can rapidly transition into a disease state^61^. In the disease state, the intracellular oxidation reaction is no longer elevated, but cells have undergone oxidative modification, resulting in a relatively stable but challenging-to-treat state. Thus, intracellular lipid peroxidation products and oxidative modification of genes and proteins may serve as crucial characteristics of disease-susceptible state and merit further investigation (Fig. 2B).

## 5 Mathematical modeling and artificial intelligence for disease susceptibility detection and warning

**Figure 3:**
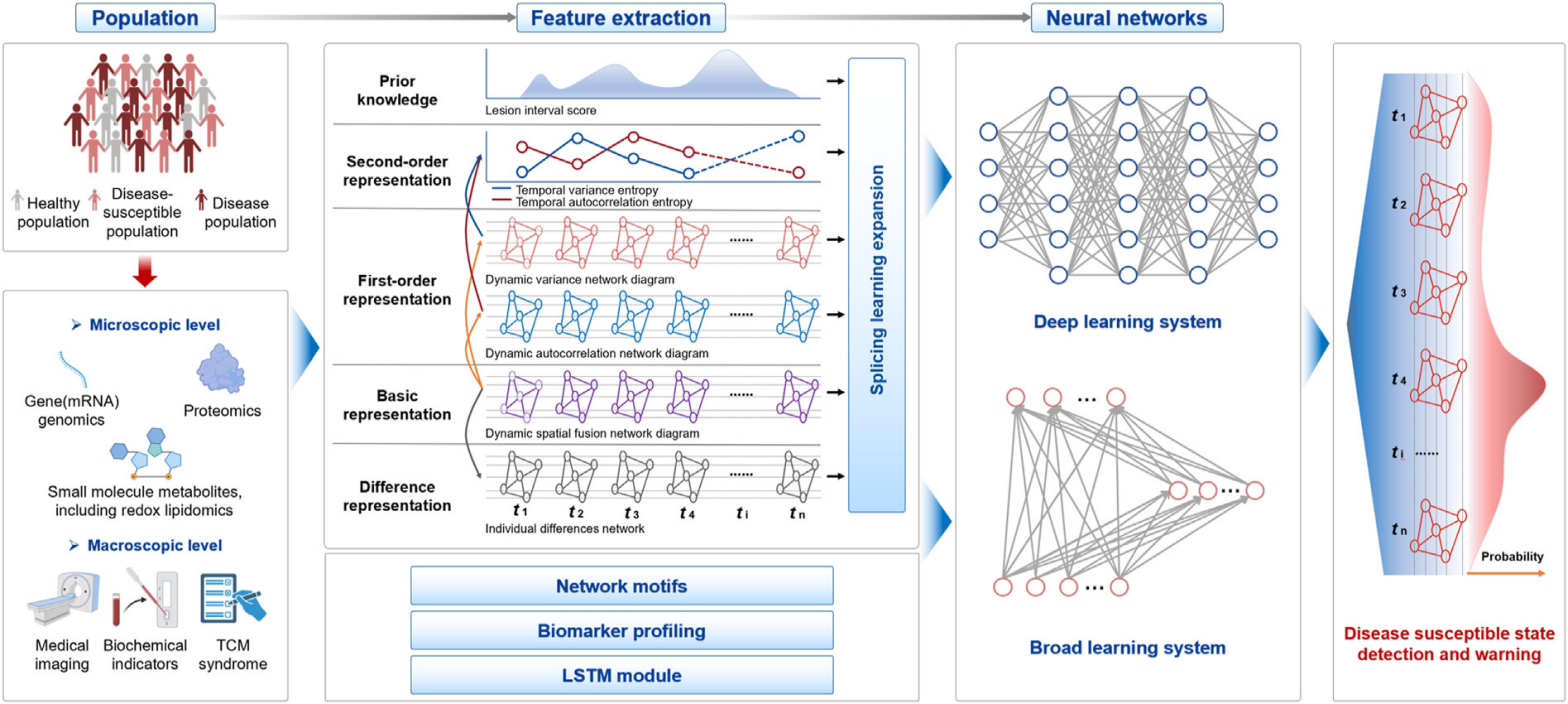
The proposed framework for identifying disease-susceptible state. This framework integrates macroscopic and microscopic data from multiple time windows using a splicing learning algorithm to augment the dataset for model training. The process begins with constructing a dynamic spatial fusion network as the foundational representation, followed by the development of dynamic variance and autocorrelation networks to form first-order representations. These are further processed into second-order representations, such as temporal variance entropy and temporal autocorrelation entropy. A long short-term memory (LSTM) network is then used to extract low-dimensional feature mappings. The resulting features are further analyzed by deep learning or broad learning systems to detect disease-susceptible states. The results are aggregated across time windows to determine the probabilities of health, disease susceptibility, and disease states. Several elements in the figure are sourced from BioRender.com.

The early warning and prediction of disease-susceptible states are crucial for the implementation of preventive strategies in medicine. Traditional clinical diagnostics, which primarily rely on imaging, biochemical analysis, and pathology, often face significant challenges in distinguishing the critical states from normal conditions. The rapid advancements in artificial intelligence (AI) especially on machine learning (ML) present promising tools to address these challenges. These methods do not rely on explicitly programmed instructions but instead leverage data-driven algorithms to identify patterns and make predictive decisions. Predictive models analyze both longitudinal data (such as sequential clinical assessments, medical imaging, and laboratory diagnostics) and static information (such as the patient’s general characteristics, including age, ethnicity, and genetic markers) to assess current health status and forecast future health outcomes^62^. Within the realm of AI, various new predictive models can be proposed, *via* generative intelligence or decision intelligence methods. Traditional ML algorithms, such as decision trees and support vector machines, often require the identification and directly selection of relevant features from data, making them suitable for small sample sizes. In contrast, DL, such as convolutional neural networks (CNN), recurrent neural networks (RNN), and transform, employs multi-layer neural networks to automatically extract features without manual intervention, excelling in handling large-scale and high-dimensional datasets^63^, ^64^, ^65^. While traditional machine learning models excel at handling static data, they have limitations in capturing temporal dependencies and dynamic changes within data, a shortcoming effectively addressed by the temporal analysis capabilities of models such as long short-term memory (LSTM). Actually, deep learning-based models have shown potential in addressing these challenges by accurately forecasting disease progressions such as cardiac arrest, respiratory failure, cancer, and depression^66^, ^67^, ^68^. The application of deep learning uncovered effective features for predicting patient survival, providing new perspectives for biomarker discovery and enhancing clinical decision-making frameworks^69^. Additionally, biomarkers and disease can also be effectively recognized using the latest techniques such as GAN, LLM, and CGMformer models^70^, ^71^, ^72^. Building on advancements in predictive models and algorithms, we propose a system that integrates temporal and neural networks to predict and diagnose health states. This model employs dynamic space fusion networks and LSTM for multi-level feature mapping, constructing a robust framework for early detection and intervention. By integrating macroscopic and microscopic data from multiple time windows, the model enhances the prediction accuracy of health, disease susceptibility, and disease states (Fig. 3). It serves as a powerful tool in the development of TCM and herbal intervention strategies, providing a robust framework for early detection and timely treatment.

**Figure 4:**
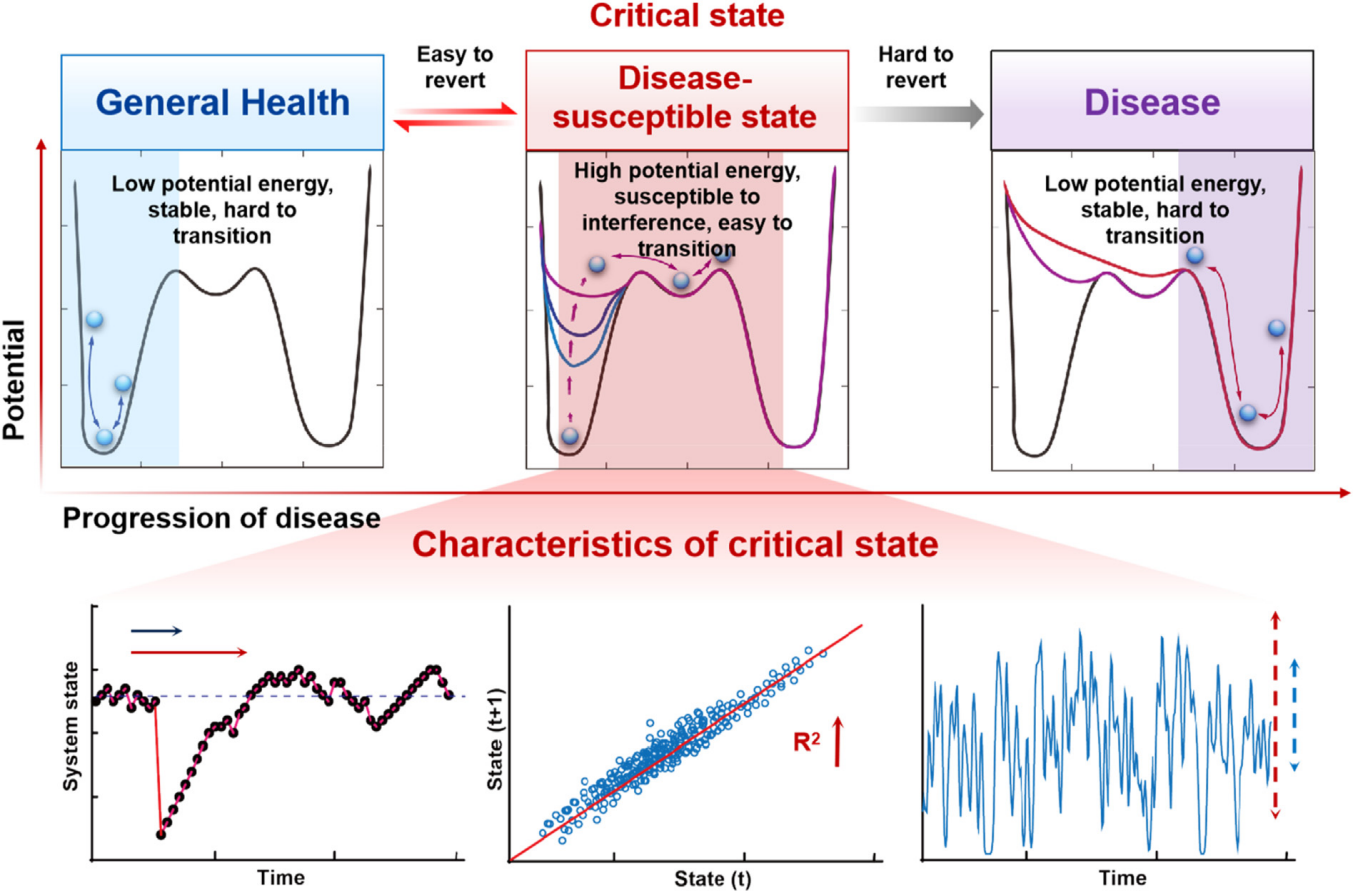
The characteristics of critical state during the health→disease-susceptible state→disease progression. The healthy state is defined as the state of the system with low local potential energy, high stability, strong resilience, and a robust ability to resist external interference. In contrast, the disease-susceptible state represents the normal limit state of the system with high potential energy, poor stability, low resilience, and susceptibility to external interference, but still reversible. The disease refers to a stable state of the system with low resilience, difficult to interfere, and challenging to reverse. The disease-susceptible state is characterized by reduced elasticity and a reduced recovery rate when disturbed. Additionally, the autocorrelation coefficients of fluctuations in this critical state are increased, and there are increased stochastic fluctuations in these states. These characteristics have been identified in the relevant literature^74^^,^^91^.

The construction of diagnostic models critically depends on extensive continuous clinical data and often suffers from limited interpretability. This has driven researchers to explore high-dimensional omics data to identify biomarkers capable of characterizing disease-susceptible states. A promising approach in this filed is the identification of the “critical slowing down” (CSD) phenomenon observed during disease progression^73^. CSD is a phenomenon observed in complex systems across various disciplines, such as physics, biology, and economics, indicating that a system is approaching a tipping point. It is characterized by a decreased recovery rate from perturbations, which can be detected or inferred from increased lag-1 autocorrelation and variance^74^ (Fig. 4). This discovery has sparked the development of mathematical models designed to capture the universal features of disease-susceptible states. Dynamic network biomarkers (DNB) have emerged as a model-free approach for the early warning of the critical state of disease^75^, ^76^, ^77^, ^78^. By utilizing the principles of nonlinear dynamics and bifurcation theory, DNBs identify functional modules within complex biological networks that undergo dynamic changes over time, rather than relying on a static set of biomolecules. The effectiveness of DNB has been demonstrated in various diseases such as influenza outbreaks, tumor progression, and diabetes^79^, ^80^, ^81^, ^82^, ^83^, ^84^, ^85^, ^86^. Another notable approach in this field is the UNIQ system, which represents a pioneering method for studying critical disease biomarkers. Rooted in the holistic principles of TCM, the UNIQ system^87^ integrates the concept of “taking the image and comparing the category” at a microscopic level. This system utilizes a “macro-micro modular correlation” approach, linking the human phenotype network (including clinical manifestations of TCM syndromes) with the biomolecular network (genes, proteins), and drug networks. By objectively and quantitatively describing these relationships, the UNIQ system facilitates a comprehensive decoding of the interactions between diseases, drugs, genes, and proteins. The system has been particularly effective in predicting early biomarkers for diseases like gastric cancer, facilitating earlier interventions and highlighting the potential of TCM in modern medicine^88^. Moreover, researchers have developed individual-specific methods such as the sample-perturbed network entropy method^77^, utilizing high-dimensional omics data rich in physiological and pathological features of individual samples, which opens new avenues for personalized and precise prediction. Similarly, gene regulatory network is valuable for predicting critical states by modeling gene interactions, where gene expression is regulated by upstream genes and affects downstream targets. The expression values, defined as gene vectors, can characterize the state of a sample, thereby predicting the transition state^89^^,^^90^. However, translating these biomarker discoveries into clinical practice remains challenging, primarily due to the complexities of omics technologies and the extensive foundational research required. Despite considerable progress in predictive modeling and early detection of diseases, the development of effective models capable of accurately identifying disease progression remains an ongoing challenge. Addressing issues such as small sample sizes, high-dimensional data, and complex data environments will be crucial in advancing this field.

## 6 Prospects

The disease-susceptible state is characterized by its dynamic, multi-dimensional, complex, systemic, and deeply influenced by individual variability. This complexity raises a series of scientific questions that need to be addressed in the modern research of Weibing theory and its clinical practice. These include: 1) The identification of the biomedical basis of Weibing and the key effector molecules driving the development of disease; 2) The establishment of an objective indicator that can quantitatively characterize the state of disease susceptibility; 3) The formation of early warning, prediction methods, and intervention approaches for the disease-susceptible state. To tackle these pressing questions, interdisciplinary collaboration is paramount, informed by the ancient wisdom of traditional Chinese medicine’s “prevention of disease” philosophy. This necessitates the seamless integration of medicine, biology, mathematics, artificial intelligence, and other disciplines, fostering a holistic understanding and innovative solutions. We are confident that with unwavering dedication and continuous endeavors, we can fully exploit the profound implications of the “prevention is better than cure” mindset and usher in a novel paradigm of scientific research centered on “disease prevention” within the framework of TCM.

## Author contributions

Rong-Rong He, Hao Gao, Hiroshi Kurihara, and Xin-Sheng Yao conceived the project. Wan-Yang Sun, Rong Wang, and Rong-Rong He drafted the original manuscript. Shu-Hua Ouyang, Junwei Duan, Wan-Li Liang, Wen-Yong Gong, Lian-Ting Hu, Xiu-Juan Chen, and Yi-Fang Li collected and analyzed data. Rong-Rong He and Hao Gao revised the manuscript. All the authors read and approved the final manuscript.

## Conflicts of interest

The authors declare no conflicts of interest.

## Appendix A Supporting information

The following is the Supporting Information to this article:
