# Supplementary material for: “Weibing” in traditional Chinese medicine—biological basis and mathematical representation of disease-susceptible state
Source: Acta Pharm Sin B. 2025 Mar 8;15(5):2363–71. doi: 10.1016/j.apsb.2025.03.009 (PMC12145064; doi:10.1016/j.apsb.2025.03.009)
Supplement: Multimedia component 1 [file mmc1.pdf]

Supporting Information for

Review

"Weibing" in traditional Chinese medicine—biological basis and mathematical representation of disease-susceptible state

Wanyang Sun[a,†], Rong Wang[a,†], Shuhua Ouyang[a], Wanli Liang[a,b], Junwei Duan[d], Wenyong Gong[c], Lianting Hu[e,f], Xiujuan Chen[e], Yifang Li[a], Hiroshi Kurihara[a,*], Xinsheng Yao[a,*], Hao Gao[a,*], Rongrong He[a,b,*]

[a]*Institute of Traditional Chinese Medicine and Natural Products, College of Pharmacy/Guangdong Engineering Research Center of Traditional Chinese Medicine & Disease Susceptibility/Guangdong Engineering Research Center of Traditional Chinese Medicine & Health Products/International Cooperative Laboratory of TCM Modernization and Innovative Drug Development of Chinese Ministry of Education (MOE)/Guangdong Province Key Laboratory of Pharmacodynamic Constituents of TCM and New Drugs Research/State Key Laboratory of Bioactive Molecules and Druggability Assessment, Jinan University, Guangzhou 510632, China*

[b]*State Key Laboratory of Quality Research in Chinese Medicine, Macau University of Science and Technology, Macau 999078, China*

[c]*College of Information Science and Technology, Jinan University, Guangzhou 510632, China*

[d]*Faculty of Data Science, City University of Macau, Macau 999078, China*

[e]*Medical Big Data Center, Guangdong Provincial People's Hospital, Guangdong Academy of Medical Sciences, Guangzhou 510632, China*

[f]*The Data Center, Wuhan Children's Hospital (Wuhan Maternal and Child Healthcare Hospital), Tongji Medical College, Huazhong University of Science and Technology, Wuhan 430016, China*

Received 26 November 2024; received in revised form 27 February 2025; accepted 5 March 2025

*Corresponding authors.

E-mail addresses: rongronghe@jnu.edu.cn (Rongrong He), tghao@jnu.edu.cn (Hao Gao), tyaoxs@jnu.edu.cn (Xinsheng Yao), Hiroshi_kurihara@163.com (Hiroshi Kurihara)

[†]These authors made equal contributions to this work.

Words in Chinese

Weibing: 未病

Shanghuo: 上火

syndrome: 证

heat syndrome: 热证

cold syndrome: 寒证
